# Improvement in the photoelectric conversion efficiency for the flexible fibrous dye-sensitized solar cells

**DOI:** 10.1186/s11671-018-2601-7

**Published:** 2018-06-28

**Authors:** Gentian Yue, Xianqing Liu, Ying Chen, Jinghao Huo, Haiwu Zheng

**Affiliations:** 1 Henan Key Laboratory of Photovoltaic Materials, Henan University, Kaifeng, 475004 China; 2 School of Physics & Electronics, Henan University, Kaifeng, 475004 China; 3 School of Materials Science and Engineering, Shaanxi University of Science and Technology, Xi’an, 710021 China

**Keywords:** Flexible, Multilayer, Fibrous, Dye-sensitized solar cell

## Abstract

A dye-sensitized and flexible TiO_2_ fiber with multilayer structure was prepared by using brush method as the photoanode in the efficient flexible fibrous dye-sensitized solar cells (FFDSSCs) to avoid electronic recombination and improve the electronic capture efficiency. The composite Pt counter electrode, preparation from the surface modification of the electrodeposited Pt wire by using a simple one-step thermal decomposition approach of H_2_PtCl_6_ isopropanol and n-butyl alcohol (volume ratio = 1:1) solution, provided a significant improvement in electrocatalytic activity, which was confirmed by extensive electrochemical tests. The FFDSSC assembled with the fiber-shaped TiO_2_ photoanode and the composite Pt counter electrode achieves an enhanced photoelectric conversion efficiency of 6.35%, higher than that of the FFDSSC with monolayer fibrous TiO_2_ photoanode and electrodeposited Pt wire counter electrode. More importantly, the photoelectric conversion efficiency of 6.35% is comparable to that of the FFDSSC based on the pure Pt wire counter electrode (6.32%). The FFDSSC with high elasticity, flexibility, and stretchability can adapt to complex mechanical deformations, which is of great significance for the development of wearable electronics in the future.

## Background

Dye-sensitized solar cells (DSSCs) are regarded as one of the most promising next-generation photovoltaic cells to replace conventional Si-based solar cells for their advantages of low-cost, high power conversion efficiency, and environmental friendliness [1, 2]. However, the solar cells with the rigid conductive glass are now facing limitations in practical application such as transportation, installation, handling, and smart textile systems [3–5]. To overcome such issues and to broaden its application area, fiber-shaped solar cells as a promising candidate for future applications with various directions are extensively concerned by DSSC researchers.

Fibrous solar cells exhibit unique advantages of being lightweight, wearable, and adaptive to a variety of curved surfaces like our bodies in comparison with planar photovoltaic devices and thus are robustly developing to fulfill the demands of various wearable electronics in modern life [6–8]. In addition to the advantages of the flat plate flexible DSSC, fiber-like solar cells have unique advantage of three-dimensional lighting, which can make full use of diffuse light from all angles.

Several studies on fibrous DSSCs have been reported preparation with modified titanium wire as photoanode and pure platinum (Pt) wire as counter electrodes (CE) [9, 10]. Of course Pt is one of the most selective materials for catalyzing the reduction of I_3_^−^ to I^−^ due to its superior electrocatalytic activity, stability, and excellent conductivity; however, it is very high-cost with pure Pt wire as CE and disfavors the scale-up production for fibrous devices. Therefore, effective design of electrode including low-cost CE with high conductivity and catalytic ability is essential. Many reports have discovered several options by thermal decomposition or electrochemical reduction to prepare the Pt films with the same function as pure platinum, which significantly reduces the usage amount of platinum [11–14]. In addition, the performance of the FFDSSC with modified titanium wire photoanode is low due to its small loading of dye and electron recombination. Many attempts are made to enhance the efficiency of the optical absorption and charge transport by surface modification, particle size changing, and multi-structure construction for the photoanode. The main aim is to develop high-performance fiber electrodes in enhancing the photovoltaic performances of fiber-shaped solar cells.

Herein, a flexible fibrous DSSC (FFDSSC) based on a flexible fibrous TiO_2_ photoanode with smooth surface coated on the Ti wire substrate by using a multi-step sintering method, and a modified Pt composite CE prepared with Al wire as inner core via a two-step electrochemical-thermal decomposition approach were envisaged to enhance the photo-electric conversion efficiency. As expected, the modified composite Pt CE showed excellent electrocatalytic activity and low charge transfer resistance of 3.11 Ω cm^2^ through the extensive electrochemical measurements. The FFDSSC exhibited a considerably improved performance in photo-electric conversion efficiency of 6.35% under irradiation of 100 mW cm^−2^ (AM 1.5).

## Methods

### Materials

The nickel (II) chloride hexahydrate (NiCl_2_·6H_2_O, 98%), thiourea (TU, ≥ 99.0%), cobalt chloride hexahydrate (CoCl_2_·6H_2_O, 98%), ethanol, chloroplatinic acid, titanium tetrachloride (TiCl_4_), and tetra-n-butyl titanate are purchased from Shanghai Chemical Agent Ltd., China. All reagents are of analytical reagent grade. The aluminum and titanium wire (diameter = 0.2 mm, 99.999%) are purchased from Shengshida Metallic Material Co., Ltd. China. The organometallic compound sensitized dye N719 is obtained from Solaronix SA (Switzerland). The TiO_2_ paste (diameter = 20 nm) are purchased from Wuhan Geao Co., Ltd. China.

### Preparation of flexible fibrous TiO_2_ photoanode

The aluminum and titanium wires with length of 15 cm were polished by sandpaper and ultrasonically cleaned sequentially in detergent, acetone, distilled water, and ethanol for 30 min, respectively, and then stored in isopropyl alcohol. The TiCl_4_ solutions were configured with the concentration of 0.03 and 0.05 M and stored in refrigerator.

The dye-sensitized flexible fibrous TiO_2_ photoanode was prepared referred to our previous reports [15–17]. Firstly, a barrier layer was formed by immersing the titanium wire substrate with length of 15 cm in 0.03 M TiCl_4_ solution at 70 °C for 1 h, followed by sintering at 450 °C for 30 min in air. This process repeats five times to increase the loading of TiO_2_. Subsequently, the TiO_2_ layer with particle size of 20 nm was coated onto the barrier layer by using brush method and then sintered at 450 °C for 30 min in air. This process repeats three times to form a smooth surface. A modified layer is formed by immersing the abovementioned TiO_2_ substrate in 0.05 M TiCl_4_ solution at 70 °C for 1 h and sintering at 450 °C for another 30 min. This process repeats two times to make sure the TiO_2_ pores are filled. The dye was loaded by immersing the fibrous TiO_2_ anode in 0.3 mM of dye N719 Tert-butanol/acetonitrile solution for 12 h. Thus, the dye-sensitized flexible fibrous TiO_2_ photoanode was obtained.

### Preparation of Pt fibrous CE and fabrication of FFDSSCs

**Fig. 1:**
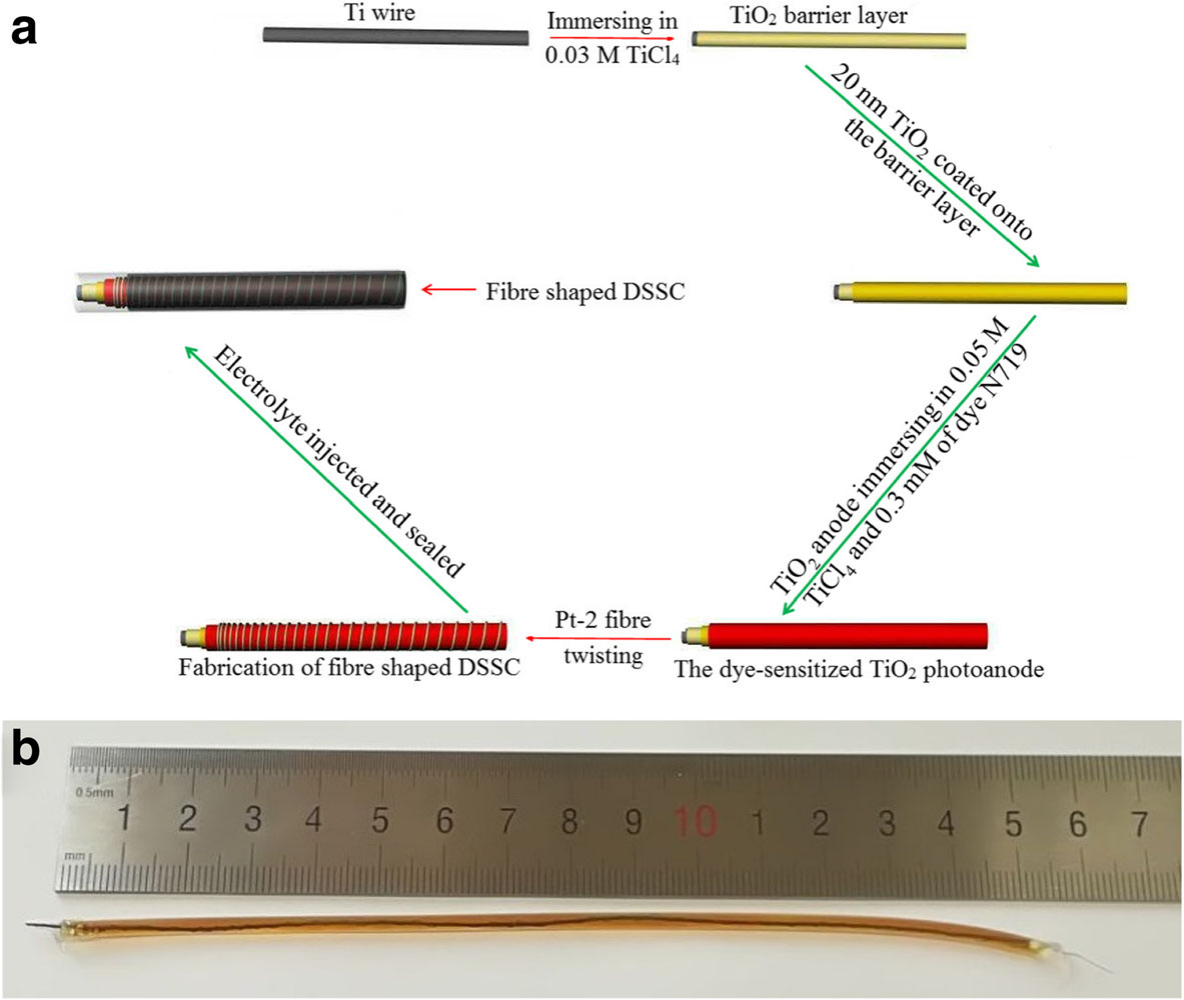
Schematic illustration of the fiber-shaped DSSC fabrication. **a** Fabrication process of the fiber-shaped DSSC. **b** Photograph of the fiber-shaped DSSC

Fibrous Pt CE was prepared by a two-step electrochemical-thermal decomposition approach. Firstly, aluminum wire was soaked into 0.01 M H_2_PtCl_6_ and LiClO_4_ ethanol solution to carry out the electrodeposition procedure and got Pt-1 fibrous CE. The obtained Pt-1 fibrous CE was heated to 250 °C, and then 0.5 ml of H_2_PtCl_6_ (1.0 wt%) isopropanol and n-butyl alcohol (volume ratio = 1:1) solution containing OP emulsification agent (1.0 wt%) was rapidly dropped onto the surface of the Pt-1 fibrous CE and sintered at 450 °C for 30 min to remove some residual organic compounds in the platinum layer, and a microporous platinum film thus was prepared and signed Pt-2 fibrous CE. The Pt-2 fibrous CE was twisted around the fibrous TiO_2_ photoanode with approximately 0.5 mm pitch to form a flexible fiber-shaped DSSC (as shown in Fig. 1). The resulted wire was sealed into a plastic tube (diameter of 0.5 mm), and redox electrolyte (0.05 M of I_2_, 0.1 M of LiI, 0.6 M of tetrabutylammonium iodide, and 0.5 M of TBP in acetonitrile) was injected with a syringe and sealed with the UV-cure adhesion (HT8803) to prevent from leakage or evaporation of liquid electrolyte. For comparison, the flexible fiber-shaped DSSCs (based on the Pt-1 and pure Pt CEs, and the fibrous TiO_2_ photoanode with and without TiCl_4_ modification) were prepared using a similar process.

### Characterization

The surface morphologies of the samples were observed by using JSM-7001F field emission scanning electron microscope (SEM). Energy dispersive spectroscopy analysis (EDS) was obtained from Bruker-ASX (Model Quan-Tax 200). Cyclic voltammetry (CV) measurements were conducted in a three-electrode one-compartment cell, in which an as-prepared Pt wire was taken as the working electrode, a Pt sheet of 1.5 cm^2^ as CE, and an Ag/AgCl electrode as reference electrode in an acetonitrile solution consisting of 10 mM LiI, 1 mM I_2_, and 0.1 M LiClO_4_. The EIS tests were carried out simulating open-circuit conditions at ambient atmosphere by using an electrochemical measurement system (CHI660E, Shanghai Chenhua Device Company, China) at a constant temperature of 20 °C with AC signal amplitude of 20 mV in the frequency range from 0.1 to 10^5^ Hz at 0 V DC bias in the dark.

The photovoltaic tests of FFDSSCs were carried out by measuring photocurrent-photovoltage (J-V) characteristic curves under irradiation of 100 mW cm^− 2^ from the solar simulator (CEL-S500, Beijing China Education Au-light Co., Ltd) in ambient atmosphere. The fill factor (FF) and the photo-electric conversion efficiency (*η*) of DSSC were calculated according to the following equations:

1 $$ \upeta\ \left(\%\right)=\frac{\mathrm{Vmax}\times \mathrm{Jmax}}{\mathrm{Pin}}\times 100\%=\frac{\mathrm{Voc}\times \mathrm{Jsc}\times \mathrm{FF}}{\mathrm{Pin}}\times 100\% $$

2 $$ \mathrm{FF}=\frac{V\max \times J\max }{V\mathrm{oc}\times J\mathrm{sc}} $$

where *J*_sc_ is the short-circuit current density (mA cm^−2^); V_oc_ is the open-circuit voltage (V), *P*_in_ is the incident light power (mW cm^−2^) and *J*_max_ (mA cm^−2^) and *V*_max_ (V) are the current density and voltage at the point of maximum power output in the J–V curve, respectively.

## Results and discussion

### Surface morphology and composition of the samples

**Fig. 2:**
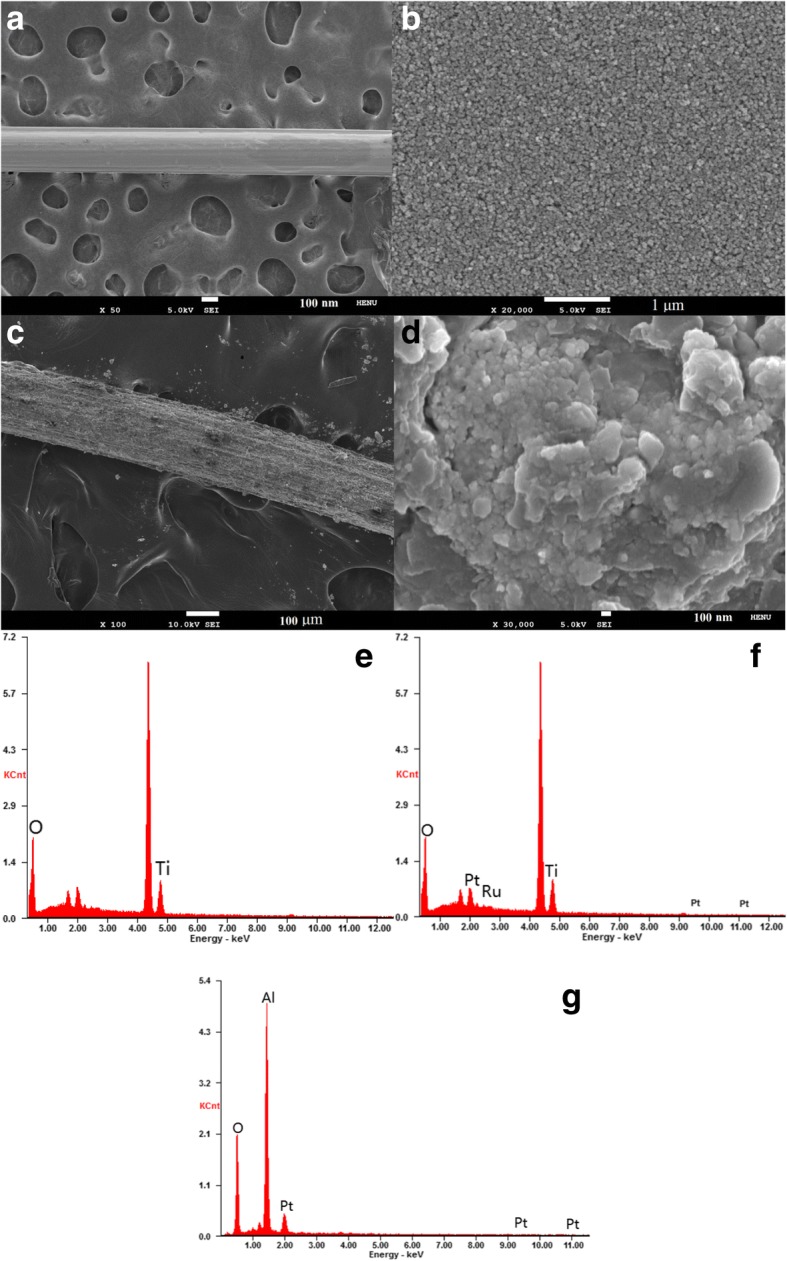
The SEM images of the TiO_2_ photoanode (**a**, **b**) and fibrous Pt CE (**c**, **d**) with different resolutions, the EDS images of the TiO_2_ photoanode before (**e**) and after (**f**) sensitizing, and the fibrous Pt CE (**g**)

Figure 2 presents the SEM images of the fiber-shaped TiO_2_ photoanode and Pt CE with different resolutions, the EDS images of the fibrous Pt CE, and the TiO_2_ photoanode before and after sensitizing. From Fig. 2a and b, the fiber-shaped TiO_2_ photoanode remains smooth surface and porous structure, and the TiO_2_ nanoparticles disperse uniformly in the Ti wire. Thus, the fiber-shaped TiO_2_ photoanode modified with TiCl_4_ twice formed the TiO_2_ barrier layer, which can effectively prevent the electron recombination between the electrolyte and Ti fiber. From Fig. 2c and d, it can be seen that the surface of the fibrous Pt CE is smooth, uniform micropores and few bulges, which came from the rapidly boiling and volatilizing of isopropanol, and a large amount of pores were in situ generated in the surface of the electrodeposition Pt. Such a surface morphology of the modified fiber-shaped Pt CE largely increases the specific surface area of platinum fiber and are availed for the adsorption of liquid electrolyte [18], consequently resulting in a great improvement of photocurrent density and open-circuit voltage for the FFDSSC. Figure 2e and f show the EDS images of the TiO_2_ photoanodes before and after sensitizing, respectively. Compared to Fig. 2e, f reveals that the TiO_2_ photoanode was successfully sensitized from the strong signal of Ru element. The strong signals for the Al and Pt elements as shown in Fig. 2g indicate that the fibrous Pt CE was prepared with Al wire as inner core.

### Electrochemical properties

**Fig. 3:**
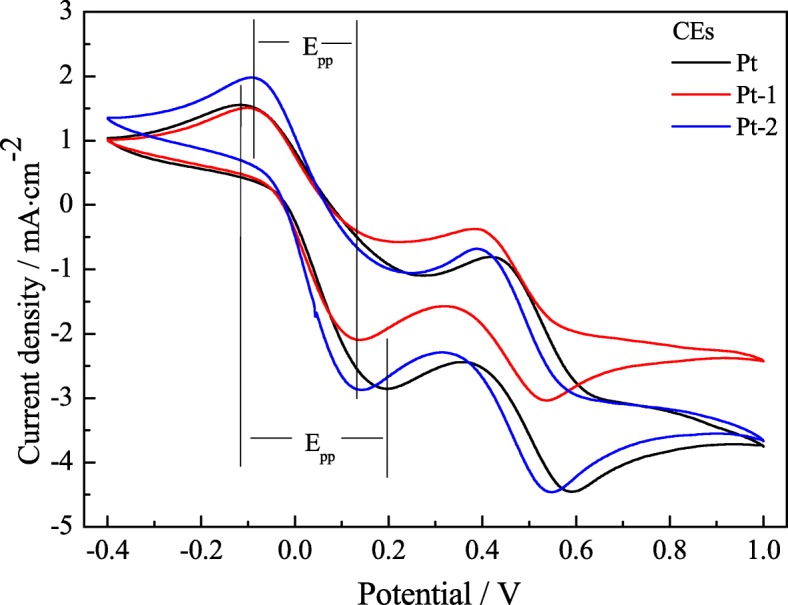
The cyclic voltammograms for the Pt-1, Pt-2, and pure Pt CEs at the scan rate of 50 mV s^−1^

Figure 3 presents the cyclic voltammograms of the electrodeposition Pt CEs before and after modified with thermal decomposition Pt at the scan rate of 50 mV s^−1^ to investigate the electrocatalytic activity of the samples in the potential interval of − 0.4 to 1.0 V. To our knowledge, the absolute value of cathodic peak current density |I_pc_| is positively correlated with the catalytic ability of electrodes, and the absolute value of peak-to-peak separation |E_pp_| is inversely correlated with the electrocatalytic activity of the CEs [19, 20]. Figure 3 gives almost identical shapes of two pairs of redox peaks and |E_pp_| for the Pt-1 and Pt-2 CEs in I^−^/I_3_^−^ redox system, and the |I_pc_| of the Pt-1 and Pt-2 CEs are 2.10 and 2.87 mA cm^−2^, respectively, which exhibits much higher cathodic peak current density for the Pt-2 CE. This attributes to the large active surface area and micropores structure of the Pt-2 CE made from the rapidly boiling and volatilizing of isopropanol, and a large amount of pores generated in situ in the platinum film. It was noticed that though the Pt-1 and Pt-2 CEs exhibit a similar |E_pp_|, however, the Pt-2 CE shows much higher |I_pc_| than that of the Pt-1 CE. This indicates that the Pt-2 CE is more effectively acted as a catalyst in the reaction of the I^−^/I_3_^−^ electrolyte than that of the Pt-1 CE. More importantly, the Pt-2 CE with double-layer structure exhibits higher values for the |I_pc_| and |E_pp_| than that of the pure Pt fiber CE (listed in Table 1). The fact fully proves that the Pt-2 CE with low-cost and simple preparation performs the same function as the pure Pt fiber CE. Consequently, the electrodeposition Pt CE modified with thermal decomposition Pt is an efficient electrocatalyst and has a good electrocatalytic ability for the I^−^/I_3_^−^ redox reaction.

**Table 1:** Cyclic voltammograms and EIS parameters of the various CEs

No.	CE	*R*_*s*_ (Ω cm^2^)	*R*_ct_ (Ω cm^2^)	*Z*_*w*_ (Ω cm^2^)	|E_pp_| (v)	|I_pc_| (mA cm^−2^)
a	Pt	3.75	3.10	1.62	0.310	2.85
b	Pt-1	3.96	3.99	1.09	0.231	2.10
c	Pt-2	3.57	3.11	1.05	0.230	2.87

**Fig. 4:**
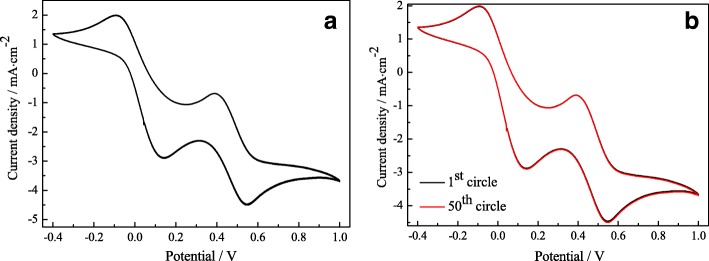
Cyclic voltammograms for the Pt-2 CE at the scan rate of 50 mV s^−1^. 50 cycles continuous scanning (**a**); the cyclic voltammograms of the 1st and 50th circle (**b**)

Figure 4 shows 50 cycles cyclic voltammograms for the Pt-2 CE at the scan rate of 50 mV s^−1^ to investigate the long-term electrochemical stability of a CE. As presented in Fig. 4, the normalized cathodic and anodic peak current densities keep scarcely changed after being tested for 50 consecutive cycles. This suggests that the electrodeposition Pt CE after modified with H_2_PtCl_6_ thermal decomposition coated on the Al substrate possesses excellent electrochemical and chemical stability.

**Fig. 5:**
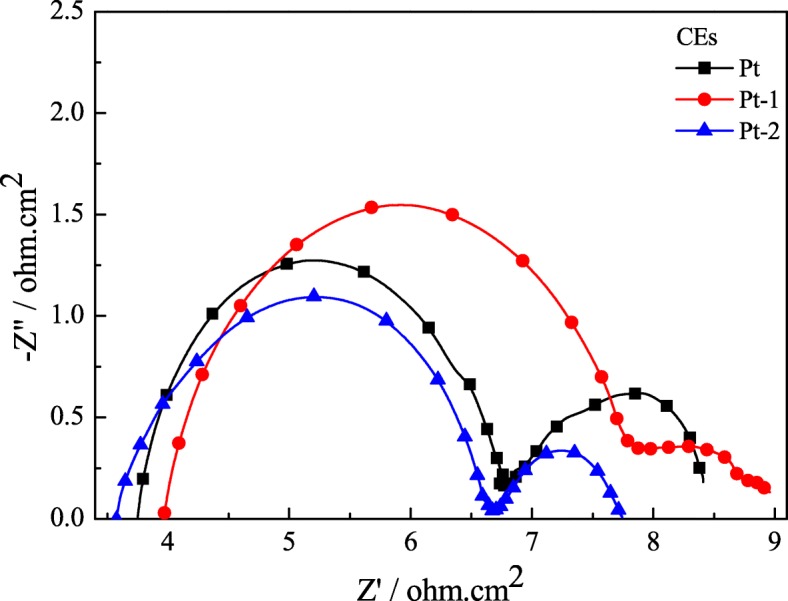
EIS of the Pt-1, Pt-2, and pure Pt CEs for I^−^/I_3_^−^ redox couple

Electrochemical impedances of the CEs are effective and extensive tools to investigate the charge transport process. Figure 5 shows the Nyquist plots of the symmetrical Pt-1, Pt-2, and pure Pt CEs for I^−^/I_3_^−^ electrolyte, and the corresponding EIS parameters are also listed in Table 1, in which the *R*_*s*_ is the resistance value at the onset point of the first semicircle, the *R*_ct_ is the radius of the first semicircle, and the semicircle represents the Nernst diffusion impedance (*Z*_*w*_) corresponding to the diffusion resistance of the I^−^/I_3_^−^ redox species [21, 22]. As is known to all, the *R*_ct_ is a crucial parameter for comparing the electrocatalytic ability of different CEs, which is inversely correlated with the catalytic ability of the CEs. From Fig. 5 and Table 1, the *R*_*s*_ associated with the Pt-1, Pt-2, and pure Pt CEs are 3.96, 3.57, and 3.75 Ω·cm^2^, respectively. The *R*_ct_ for the Pt-1, Pt-2, and pure Pt CEs are 3.99, 3.11, and 3.10 Ω cm^−2^, respectively. In other words, the *R*_*s*_ and *R*_ct_ for the abovementioned CEs follow the orders of Pt-1> Pt-2 > Pt. Thus, it is comparable to that of the Pt-1 CE and indicates a lower interfacial charge transfer resistance occurred at the interface between the Pt-2 CE and I^−^/I_3_^−^ electrolyte under the same testing conditions. These results fully prove that the Pt-2 CE with double-layer structure after thermal decomposition Pt modifying shows great improvement in electrochemical catalytic ability compared to the pure Pt CE. The reasons for enhancing the performance of the CE can be attributed to the surface structure, i.e., uniform micropores and few bulges, and good electrochemical properties, which can make the electrons transmit across the Pt-2 film|Al interface easily. Based on the comprehensive consideration of EIS data, it can be prospected that the property of the Pt-2 CE is advantageous to improve the photovoltaic performance of the FFDSSCs.

**Fig. 6:**
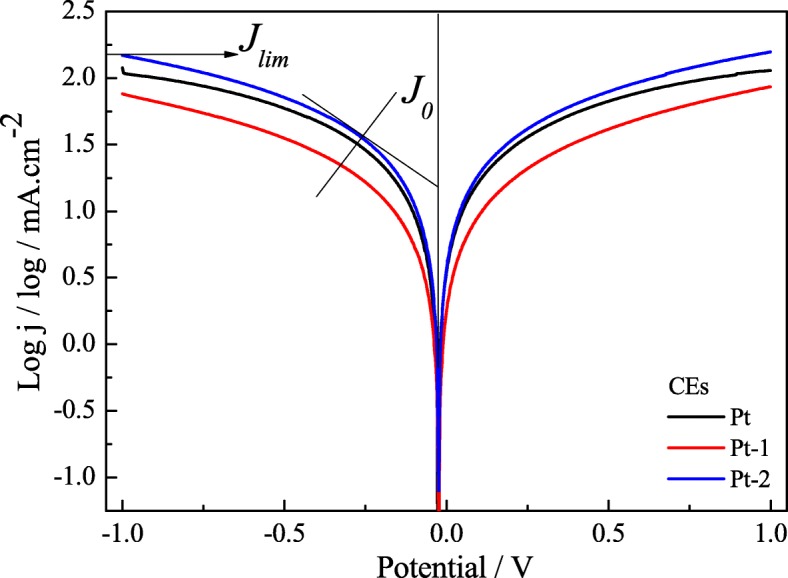
Tafel curves of the symmetrical Pt-1, Pt-2, and pure Pt CEs for I^−^/I_3_^−^ redox couple

Figure 6 presents the Tafel curves for the symmetrical cells similar to the ones used in the EIS measurements to reconfirm the electrocatalytic activity of the Pt-1, Pt-2, and pure Pt CEs. As seen in Fig. 6, the Pt-2 CE shows much larger exchange current density (*J*_0_) and limiting diffusion current density (*J*_lim_) (1.48 and 2.18 mA cm^− 2^) in comparison with that of the Pt-1 CE (1.28 and 1.89 mA cm^−2^), suggesting higher conductivity and electrocatalytic ability for the Pt-2 CE. Also, the higher *J*_lim_ for the Pt-2 CE reflects faster diffusion velocity for the redox couple in the electrolyte [23–25]. Additionally, as expected, the electrocatalytic activity of the Pt-2 CE is shown as excellent as the pure Pt CE. These positive factors can be attributed to the same reasons as CV and EIS, which logically result in an efficient power conversion efficiency for the FFDSSC. In theory, *J*_0_ is inversely proportional to *R*_ct_ according to Eq. (5) [26, 27]. The changed tendency of *J*_0_ in Tafel curves for the Pt-1, Pt-2, and pure Pt CEs is generally in accordance with the EIS. Generally, the extensive electrochemical measurements results (CVs, EIS, and Tafel) indicate that the Pt-2 CE possesses an enhanced electrocatalytic activity compared to that of the pure Pt CE; thus, it can be logically expected a considerable improvement in the photovoltaic performance for the FFDSSC.

5 $$ {J}_0=\frac{RT}{nFR_{\mathrm{ct}}} $$

where *R* is the gas constant, *T*, *F*, *n*, and *R*_ct_ have their usual meaning.

### Photovoltaic performance of the FFDSSCs

**Fig. 7:**
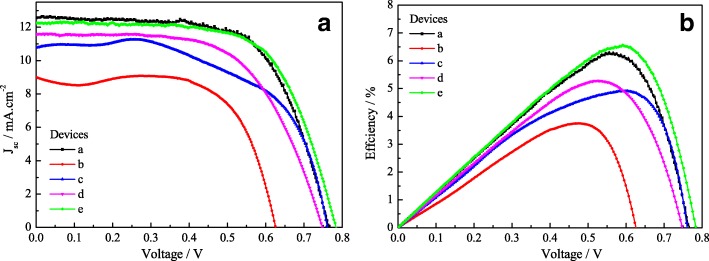
Photovoltaic performance curves for the FFDSSCs fabricated with various photoanodes and the CEs under the standard illumination. J-V characteristics of the FFDSSCs (**a**); the relationship between power conversion efficiency and the open-circuit voltage (**b**)

J-V characteristics of the FFDSSCs with different CEs and photoanodes were measured under 100 mW cm^−2^ (AM 1.5 G) irradiation, and the results are presented in Fig. 7 and Table 2. Curves b and c present the FFDSSCs assembled with Pt-1 and Pt-2 CEs and TiO_2_ photoanode without TiCl_4_ modified in Fig. 7, which are not smooth. However, it is important to note that the open-circuit voltage (*V*_oc_) and short-circuit current density (*J*_sc_) of the FFDSSC-c (0.760 V and 10.78 mA cm^−2^) is much higher than that of the FFDSSC-b (0.625 V and 10.78 mA cm^−2^). This phenomenon is associated with the low *R*_ct_, excellent electrochemical catalytic activity and conductivity for the Pt-2 CE, and the increased contact area between the Pt-2 CE and the electrolyte [28, 29]. Curves d and e of the FFDSSCs with Pt-1 and Pt-2 CEs and TiO_2_ photoanode with TiCl_4_ modified display the smooth curves with high *V*_oc_, *J*_sc_, and fill factor (FF). Between them, the higher photoelectric performance for the FFDSSC-e is mainly originating from the lower *R*_ct_, and more excellent electrochemical catalytic activity and conductivity for the Pt-2 CE compared to that of the FFDSSC-a based on the pure Pt CE and TiO_2_ photoanode with TiCl_4_ modified (6.32%). Additionally, the more important reasons are attributed to the TiCl_4_ modification, which increases the photoelectrons generated rate by the excited dye molecules and reduces the recombination rate of the electron and Ti wire; thus, the device logically shows better *V*_oc_, *J*_sc_, and FF values. Conversely, the FFDSSC based on the TiO_2_ photoanode without modification by the TiCl_4_ shows a worse photovoltaic performance. Simultaneously, the twice modification for the CE significantly influences on the performance of FFDSSC, which increases the electrolyte loading on the CE surface, and decreases the internal resistance and dark current of the FFDSSC, thus greatly improving the *J*_sc_ values. These indicate that the twice modifications for the electrode facilitate fast electron transport at the interface between I^−^/I_3_^−^ electrolyte and the electrodes, and it also can be deduced that the FFDSSC based on the Pt-2 CE and TiO_2_ photoanodes modified with TiCl_4_ can indeed improve the charge recombination and have a more outstanding effect than other FFDSSCs.

**Table 2:** The photocurrent-voltage parameters of the FFDSSCs assembled with various photoanodes and the counter electrodes

No.	CEs	Photoanodes	*V*_oc_ (V)	*J*_sc_ (mA cm^−2^)	FF	*η* (%)
a	Pt	TiCl_4_ modified	0.766	12.55	0.657	6.32
b	Pt-1	without TiCl_4_	0.625	8.98	0.667	3.74
c	Pt-2	without TiCl_4_	0.760	10.78	0.602	4.93
d	Pt-1	TiCl_4_ modified	0.747	11.59	0.611	5.29
e	Pt-2	TiCl_4_ modified	0.783	12.25	0.662	6.35

**Fig. 8:**
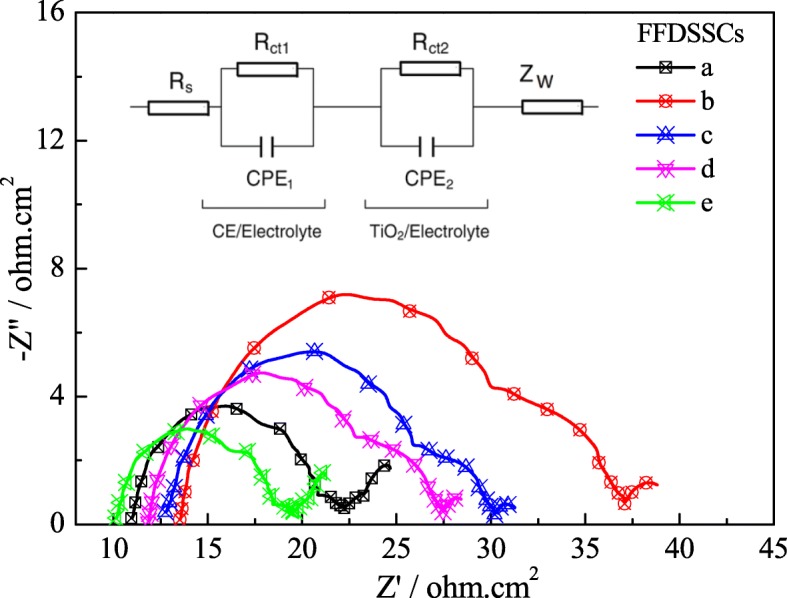
EIS for the FFDSSCs fabricated with the various photoanodes and the CEs under the standard illumination

Figure 8 shows the Nyquist plots of the FFDSSCs based on different CEs and photoanodes under 100 mW cm^−2^ (AM 1.5 G) irradiation, and the equivalent circuit is shown as the inset. *R*_*s*_ is the series resistance, and R_ct_ is the charge transfer resistance at the interface of electrolyte/photoanode. *R*_*s*_ and *R*_ct_ values for the FFDSSCs with compact layer made from TiCl_4_ modified (FFDSSCs a, d, e) are lower than those of the FFDSSCs without TiCl_4_ modified; this is due to the ultrathin TiO_2_ compact layer with a high electron mobility enhanced the interface contact between Ti wire and TiO_2_ photoanode, and it also reduces the probability of electron recombination [30, 31]. Furthermore, the FFDSSC-e possesses the smallest *R*_*s*_ and *R*_ct_ values among the FFDSSCs a, d, and e, which is smaller than that of the FFDSSC-a. This indicates that the Pt-2 CE with twice modifications in the FFDSSC is more beneficial to electron transport at the interface between I^−^/I_3_^−^ electrolyte and the electrodes than that of the pure Pt CE. As a consequence, the multiple modifications for the anode and counter electrode in FFDSSCs are conductive to improve the photovoltaic performance.

**Fig. 9:**
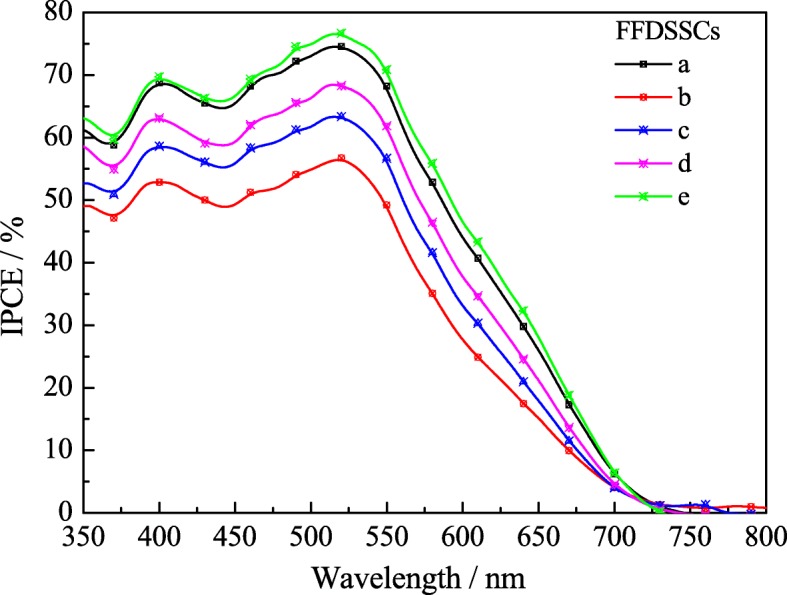
The IPCE of the various FFDSSCs

Figure 9 displays the IPCE of the FFDSSCs with different CEs and photoanodes to reflect the light response, which is directly related to *J*_sc_. As shown in Fig. 9, the maximum efficiency of all the FFDSSCs at the wavelength of about 520 nm is in coincidence with the absorption maximum wavelength of dye N719 [32, 33]. IPCE maximum peak for the above mentioned FFDSSCs follows the orders of e > a > d > c > b. This result is in good agreement with the photovoltaic performances as shown in Fig. 7, which also proves again that the multiple modifications for the anode and counter electrode can remarkably improve the photoelectric performance for the FFDSSCs.

## Conclusions

An efficient flexible fibrous dye-sensitized solar cell (FFDSSC) was fabricated with a multilayer-structure fibers TiO_2_ photoanode (modified with TiCl_4_) and a Pt-2 CE with double-layer structure to improve the performance of the device. Pt-2 fiber CE demonstrates excellent electrocatalytic activity for the reduction of triiodide in FFDSSC through the cyclic voltammetry, electrochemical impedance spectroscopy, and Tafel characterization. The FFDSSC based on the Pt-2 fiber electrode and TiO_2_ fiber photoanode modified with TiCl_4_ shows a photoelectric conversion efficiency of 6.35%, 69.8% higher than that of with monolayer fiber TiO_2_ photoanode and electrodeposition of Pt wire, which is comparable to that of the FFDSSC based on the pure Pt wire CE. This low-cost and easy fabrication FFDSSC with high elasticity, flexibility, and stretchability could prepare high-performance wearable micro-solar cells to adapt to complex mechanical deformations, which have vast potential to develop a new family in energy conversion and storage devices.

## Abbreviations

CE: Counter electrode
CV: Cyclic voltammetry
FFDSSC: Flexible fibrous dye-sensitized solar cell
I^−^/I_3_^−^: Iodide/triiodide
*J*_0_: Exchange current density
*J*_lim_: Limiting current density
*J*_ma *x*_: Maximum current density
*J*_sc_: Short-circuit current density
J-V: Photocurrent-photovoltage
PCE: Power conversion efficiency
*P*_in_: Incident light power
*R*_ct_: Charge transfer resistance
*R*_*s*_: Series resistance
SEM: Scanning electron microscopy
*V*_max_: Maximum voltage
*V*_oc_: Open-circuit voltage
